# Model validity for preclinical studies in precision medicine: precisely how precise do we need to be?

**DOI:** 10.1007/s00335-019-09798-0

**Published:** 2019-04-05

**Authors:** Abigail L. D. Tadenev, Robert W. Burgess

**Affiliations:** The Center for Precision Genetics, The Jackson Laboratory, 600 Main Street, Bar Harbor, ME 04609 USA

## Abstract

The promise of personalized medicine is that each patient’s treatment can be optimally tailored to their disease. In turn, their disease, as well as their response to the treatment, is determined by their genetic makeup and the “environment,” which relates to their general health, medical history, personal habits, and surroundings. Developing such optimized treatment strategies is an admirable goal and success stories include examples such as switching chemotherapy agents based on a patient’s tumor genotype. However, it remains a challenge to apply precision medicine to diseases for which there is no known effective treatment. Such diseases require additional research, often using experimentally tractable models. Presumably, models that recapitulate as much of the human pathophysiology as possible will be the most predictive. Here we will discuss the considerations behind such “precision models.” What sort of precision is required and under what circumstances? How can the predictive validity of such models be improved? Ultimately, there is no perfect model, but our continually improving ability to genetically engineer a variety of systems allows the generation of more and more precise models. Furthermore, our steadily increasing awareness of risk alleles, genetic background effects, multifactorial disease processes, and gene by environment interactions also allows increasingly sophisticated models that better reproduce patients’ conditions. In those cases where the research has progressed sufficiently far, results from these models appear to often be translating to effective treatments for patients.

## Validity

**Fig. 1:**
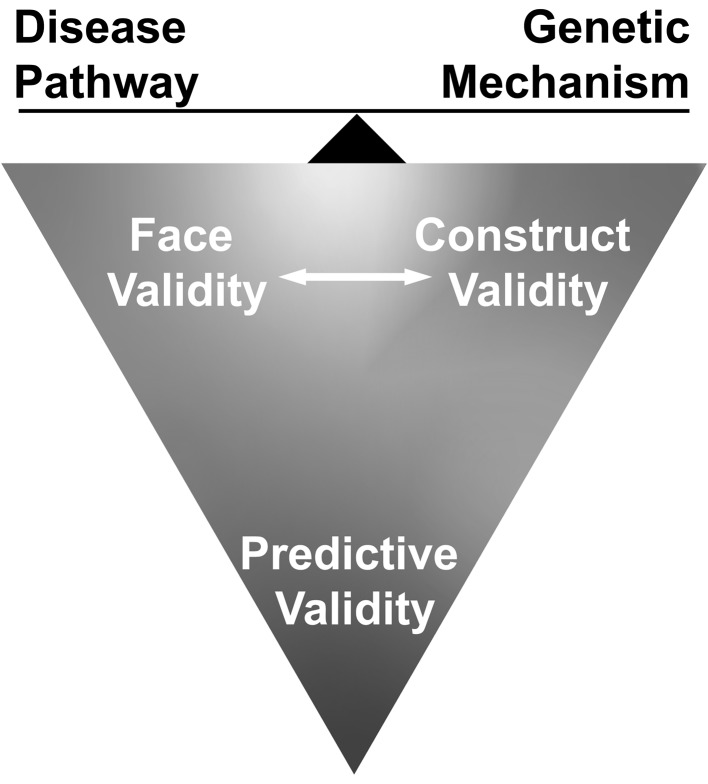
Model validity. Models can be considered for their face validity, whether they look right; their construct validity, whether they arise through the right mechanism; and their predictive validity, whether the results in the model will translate to humans. None of these measures of validity are absolutes, all are on a spectrum from strong to weak. For preclinical studies, optimizing predictive validity is critical, but whether this optimization depends on improving face or construct validity depends on context. Ideally, there is solid overlap of face, construct, and predictive validity. This is clearly preferable, but perhaps rarely completely attainable. Such overlap creates a convincing argument in preclinical studies, which are then less likely to face skepticism or questions from concerned parties such as regulatory agencies, clinicians, or patients. However, useful information can still be obtained when there is overlap only of face and predictive validity or construct and predictive validity. For example, showing that a candidate therapeutic approach is indeed relevant to a human disease requires a model with some degree of face validity. Similarly, models with strong construct validity can be used to show target engagement and other important aspects of a therapeutic approach, even if the phenotype being studied is not a perfect match to the human condition. Thus, the intended use of the model determines the relative importance of face validity/shared pathophysiology versus construct validity/shared genetic mechanism

It is important to remember that all models are models, and their limitations must be considered, as well as their potential. In evaluating models, three criteria for validity are often discussed. These include face validity: essentially, does the model look right? Does it exhibit the salient features of the condition being modeled? The second is construct validity: is the basis for the model sound? Is the condition arising for the right reasons? The third is arguably the most important for translational and preclinical research. This is predictive validity: will the results obtained with the model predict outcomes in humans? Predictive validity most often refers to drugs or treatments translating from preclinical models to clinical trials, but it also applies to more basic research on disease mechanisms such as pathophysiological responses, and whether these are equivalent in patients and the model (Fig. 1).

In genetic models, face validity becomes the phenotype and how closely it resembles the human disease. Construct validity refers to how similarly the mutation in the model recapitulates the genetic state in the patients. Ideally, changing the same conserved amino acid in a mouse protein that is changed in a human disease-associated allele causes a mouse phenotype that closely mirrors the human disease. There are certainly examples of this, but they may be the exceptions and not the rule. The reasons for this run from simple issues such as lack of conservation, to more complicated situations where gene duplications or redundancy may prevent a one-to-one recapitulation, to a lack of shared physiology or anatomy, which may or may not be known in advance, and may or may not be easily decipherable. This does not mean that the models cannot produce valuable information. To quote the statistician George Box, “Remember that all models are wrong; the practical question is how wrong do they have to be to not be useful?” (Box and Draper 1987).

In the following review, we will discuss strategies for generating models with an eye towards maximizing their predictive validity. However, in many cases, whether that has been successful is still unknown. We will also consider different measures of face and construct validity, and the extent to which their precision in reproducing the human condition may impact predictive validity. Due to personal bias and familiarity, many of the examples cited will involve mouse models of neurological and neuromuscular diseases, but the principles should extend to many disorders and many model systems, and others will be mentioned throughout.

## Monogenic diseases

Monogenic Mendelian diseases are perhaps the most straightforward starting point for this discussion. Many of the points to be made concerning face validity and construct validity as they apply to preclinical studies can be made using the example of mouse models of Nieman Picks disease type C (NPC), caused by recessive loss-of-function mutations in the *NPC1* gene. Mutations in *NPC1* were identified as the cause of NPC following the cloning of a spontaneous mouse mutation with a similar physiological and histological phenotype (Carstea et al. 1997; Loftus et al. 1997). This immediately gives credibility to the face validity of this model, as the mouse phenotype was similar enough to the human disease to allow this direct connection to be made. The NPC1 protein is a 13-transmembrane domain transporter that transports cholesterol across intracellular membranes. Loss of NPC1 function in both mice and patients leads to cholesterol accumulation in peripheral organs such as the spleen and liver, and also in neurons, leading to neurodegeneration (OMIM #257220). In mice, this neurodegeneration is most notable in cerebellar Purkinje cells, both histologically and from an early, overt ataxia. In patients, the neurodegeneration is more widespread and frequently seizures are also present, and the absence of seizures in the mouse models is a notable fault in their face validity. However, seizures may also be an aspect of the phenotype that only matters in some contexts. Managing seizures is undoubtedly an important part of the clinical care in many NPC patients. As such, research asking directly related questions such as “does this drug manage seizure activity in NPC?” or “will clearing intracellular cholesterol after the onset of the disease and neuronal loss also reduce seizure activity?” requires a model that has seizures. However, research aimed at addressing the root cause of the disease, such as gene therapy approaches to replace *NPC1* expression or strategies to clear cholesterol from cells by bypassing NPC1-mediated transport, may proceed very effectively with the mouse models as they are, given that relevant pathophysiological changes appear in relevant tissues, with similar outcomes of cholesterol accumulation and cell loss. Why the mouse models do not show seizures remains unclear, but may reflect a fundamental difference in mouse and human physiology or anatomy.

In addition to issues of face validity described above, mutations in *NPC1* also offer interesting examples of construct validity. The original mouse mutation in *Npc1* identified at NIH and an earlier spontaneous mutation, *Spm*, that was described as a disease model but never cloned (Miyawaki et al. 1982) are both truncating mutations that appear to be complete null alleles with no detectable protein produced (Maue et al. 2012). They develop very similar phenotypes (despite different genetic backgrounds of BALB/c and C57BL/6Khl) and fail to complement when inter-crossed, as expected. However, only about 20% of patients carry null alleles of *NPC1*, the rest carry a variety of point mutations, often in the “I loop” of the protein, a domain between transmembrane regions 8 and 9. Patients with point mutations tend to have later onset disease with milder (though still very severe) symptoms, suggesting a partial loss of function. Consistent with this, studies in patient lymphocytes determined that mutations in the I loop lead to an unstable or mistrafficked NPC1 protein, with levels reduced approximately 85% compared to wild type (Gelsthorpe et al. 2008). Importantly, the mutant protein retains cholesterol transport activity, suggesting that if levels could be increased, for example through “chaperone therapies” that improve trafficking through the biosynthetic pathway, function could be improved. However, such therapies cannot be tested in null alleles that produce no protein. Subsequently, a new mouse model of NPC was identified in an ENU mutagenesis program based on its phenotypes of cholesterol accumulation in the spleen and liver and cerebellar ataxia and Purkinje cell loss. Sequencing identified a single base change, converting aspartic acid 1005 in the I loop of the protein to glycine (D1005G) (Maue et al. 2012). Like the patients, these mice have a later onset and milder phenotype than the null alleles, but still a very severe disease resulting in death at 4–5 months of age. Also like the patients, these mice have NPC1 protein levels that are approximately 15% those of wild type. Therefore, this new allele is likely a better model for partial loss-of-function mutations in *NPC1*, and could enable preclinical studies for approaches such as chaperone therapies that would not otherwise be possible. The amino acid change identified in the mice does not reproduce a known human disease allele, but the amino acid is conserved, and is only two amino acids away from one of the most common human alleles, P1007A. Therefore, in terms of construct validity, the D1005G mouse is very good, and the additional precision of exactly reproducing a human disease-associated mutation may not be necessary, unless there were suspected unusual properties in the human allele.

A straightforward knockout mouse may or may not be an accurate model of a human disease, even if the disease is caused through a recessive loss-of-function mechanism. The mutations in mouse *Npc1* noted above were all identified based on phenotype; they were not engineered into the mouse genome. In that regard, their face validity may not be a surprise, but this success using phenotype driven approaches is not limited to *Npc1* and similarly valid disease models have been identified for muscular dystrophies (*Lama2*, *Chkb*), congenital myasthenic syndrome (*Agrn*), and peripheral neuropathies (*Gars*), to name just a few examples (Achilli et al. 2009; Antonellis et al. 2003; Bogdanik and Burgess 2011; Huze et al. 2009; Mitsuhashi et al. 2011; Seburn et al. 2006; Sher et al. 2006; Sunada et al. 1994; Xu et al. 1994). Since NPC results from severe loss-of-function mutations, engineered alleles such as conditional knockouts have also been successful in recapitulating the disease, and have been useful in studies separating the contributions of peripheral organs versus the central nervous system to the disease progression (Elrick et al. 2010). However, in other examples such as *Agrn*, a complete loss of function in mice results in neonatal lethality, with a complete failure of neuromuscular junction development (Burgess et al. 1999; Gautam et al. 1996). Human cases of congenital myasthenic syndrome caused by AGRN mutations are likely partial loss of function, and complete null alleles are unlikely to survive (Huze et al. 2009; Maselli et al. 2011). Mice with partial loss-of-function alleles do provide a disease model (Bogdanik and Burgess 2011). So, in cases such as *AGRN*-associated congenital myasthenic syndrome, some degree of precision and construct validity is needed, at least advancing beyond the simple approach of making a knockout to create a disease model, although the knockout phenotype does solidly implicate the *Agrn* gene in the process of neuromuscular junction formation.

Studies of *IGHMBP2* also reveal interesting issues regarding face and construct validity. The spontaneous neuromuscular degeneration (*Nmd*) mutation in mice causes early-onset motor neuron disease, and recessive mutations in *Ighmbp2* were identified as the cause (Cox et al. 1998). This finding led to the identification of human *IGHMBP2* mutations as the cause of Spinal Muscular Atrophy with Respiratory Distress (SMARD1) in humans (Grohmann et al. 2001). Interestingly, the mouse is a partial loss-of-function allele, whereas in humans, a range of alleles including likely nulls lead to disease. The mice have been used in preclinical gene therapy studies, which indicate that restoring *Ighmbp2* expression early in disease is efficacious (Nizzardo et al. 2015). However, studies in mice using tissue specific transgenic rescue indicate that if the motor neuron disease is corrected, the mice instead succumb to a dilated cardiomyopathy on a similar time course (Maddatu et al. 2004). This then raises important issues for human therapies: is rescuing motor neurons sufficient? Or do peripheral tissues such as the heart also need to be targeted? Here, determining the face validity of the mouse model with its cardiac issues may be very important, but this is currently an untested issue in patients.

However, not all monogenic diseases are straightforward to model. Two examples with varying complexity are considered below. First, Charcot-Marie-Tooth disease type IA (CMT1A) is a demyelinating neuropathy, and by far the most common form of CMT, representing nearly 57% of all cases, despite over 80 genes being implicated in CMT (DiVincenzo et al. 2014; Timmerman et al. 2014). The reason for this high frequency is the high rate of spontaneous mutation in the human genome in the region of Chromosome 17p12 (Lupski et al. 1991; Raeymaekers et al. 1991). A repeat sequence separated by 1.4 megabases is the underlying cause, and unequal crossover at the repeats leads to either duplication or deletion of the intervening sequence. This interval contains the Peripheral Myelin Protein 22 (*PMP22*) gene, encoding a protein required for peripheral myelin formation whose stoichiometry is critical (Lupski et al. 1992; Patel et al. 1992). Heterozygosity for *PMP22* results in hereditary neuropathy with pressure palsies (HNPP), whereas duplication of the gene on one chromosome leads to classic CMT1A, a similar, but clinically distinct disorder. HNPP is readily modeled in mice by loss-of-function mutations in the *Pmp22* gene (Suter et al. 1992). Indeed, the characterization of spontaneous Trembler alleles led to the identification of *PMP22* as a key dosage sensitive gene for myelination. However, the duplication seen in CMT1A does not occur spontaneously in mice, because the flanking repeats are not conserved. Nonetheless, modeling the overexpression of *PMP22* associated with CMT1A is relatively straightforward through transgenic approaches (Huxley et al. 1996; Sereda et al. 1996). Furthermore, the human gene sequence can be introduced to improve construct validity for preclinical testing of genetically based therapies, and the use of large genomic fragments such as BACs and YACs provides endogenous regulatory elements to control expression pattern and splicing. Transgene copy number can influence expression levels, but examining multiple founder lines allows this to be titered to improve face validity (Verhamme et al. 2011). This approach has been used in both mice and rats to produce very good disease models, which in turn have been used in very promising preclinical studies, in which antisense oligonucleotides were used to reduce PMP22 levels and successfully treat the disease in both rat and mouse models (Zhao et al. 2018). This preclinical study is an excellent example of using multiple models, including a model expressing the human gene, and while still untested in humans, the consistency of the results in multiple animal models bodes well for predictive validity.

**Fig. 2:**
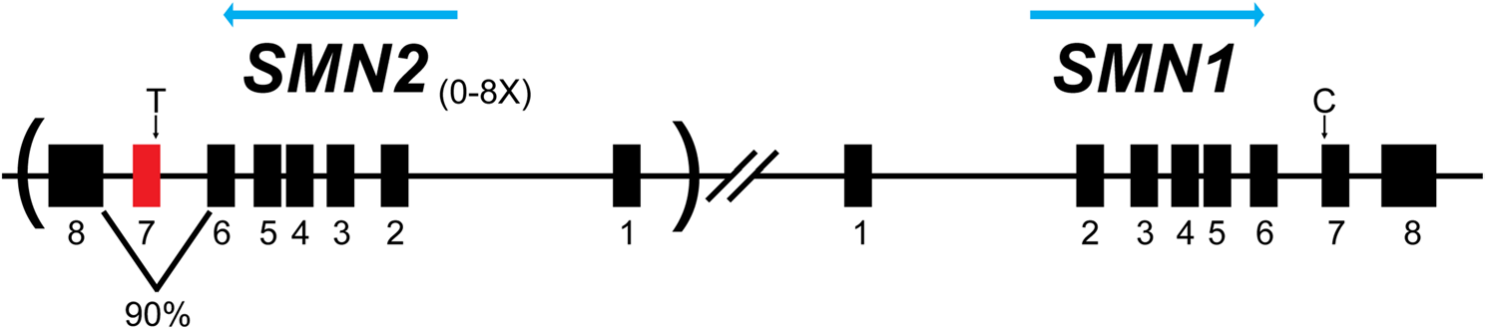
The *SMN* locus on human Chromosome 5. Virtually all cases of SMA result from a loss of function in *SMN1*. However, the inverted duplication harboring *SMN2* determines the severity of the disease. The *SMN2* gene is present with variable copy number (between zero and eight), and all copies carry a C > T transition that reduces splicing efficiency and skips exon 7 in ~ 90% of transcripts, leading to low levels of full-length protein. The duplication creating the *SMN2* gene is specific to humans, but its presence is critical to creating models that survive and have a phenotype that resembles SMA, and for testing approaches aimed at correcting its splicing and increasing the levels of full-length transcript

The second example of a more complex model of a monogenic disorder has proven to have good predictive validity in early-stage clinical trials in patients. These models are for Spinal Muscular Atrophy (SMA), a severe, early-onset motor neuron disease that is the leading genetic cause of death in infants with a carrier frequency of 1 in 54 and affecting 1 in 11,000 live births (Pearn 1978; Sugarman et al. 2012). SMA is caused by recessive mutations in the *SMN1* gene, whereas knockout of *Smn1* in mice is embryonic lethal, indicating its importance for development, but not producing a useful disease model (Lefebvre et al. 1995; Schrank et al. 1997). The increased severity in mice is because the human genome has a recent duplication event, creating a second linked locus, *SMN2* (Fig. 2). To further complicate things, the *SMN2* duplication creates variable copy numbers of the *SMN2* gene (Butchbach 2016), but the copies are not fully functional, owing to a point mutation that leads to skipping of exon seven in approximately 90% of the transcripts (Monani et al. 1999). The severity of SMA correlates with the copy number and expression levels of *SMN2*, with higher copy number producing a less severe disease (Lefebvre et al. 1997). All patients lack a functional *SMN1* gene.

The strategy to model this disease, therefore, involves creating loss-of-function mutations in mouse *Smn1*, while also transgenically expressing *SMN2*. This strategy has been generally successful, though the models tend to be quite aggressive, modeling the more severe end of the phenotypic spectrum of SMA. These models have produced useful results in defining the very early window for intervention in SMA (Lutz et al. 2011), and for studies using gene therapy vectors to replace *SMN1* (Foust et al. 2010). Indeed, even a model in pigs where a virally delivered RNAi knocks down endogenous *Smn1* was sufficient to cause a motor neuron disease. This model was then used to demonstrate that AAV9 delivery of human *SMN1*, which is not a target of the RNAi, was sufficient to rescue this disease (Duque et al. 2015). However, an alternative to restoring *SMN1* expression with gene replacement vectors is to enhance the splicing and therefore functional transcript levels of *SMN2*. Testing such strategies requires precise construct validity, introducing the human *SMN2* locus as a genomic transgene (as opposed to a cDNA, for example). Models using such constructs have been used to test both pharmacological methods and antisense oligonucleotides (ASOs) to block binding of a splice inhibitor and improve the inclusion of exon seven of *SMN2* (Hua et al. 2010; Ratni et al. 2018). These various approaches are now in clinical trials, with promising outcomes that support the predictive validity of the animal models (Finkel et al. 2017; Mendell et al. 2017; Mercuri et al. 2018), and nusinersen, the ASO promoting *SMN2* splicing, was recently approved by the US Food and Drug Administration and the European Medicines Agency as the first treatment for SMA.

## Complex diseases

As described above, models of diseases with promising predictive validity can be generated, even when the human genetic basis is not readily present in the model organism. The precise modeling of complex diseases presents a different challenge, specifically that the underlying genetic basis of the disorder is usually not fully understood in humans, and gene by environment interactions are also often involved. Alzheimer’s disease (AD) offers one example of this challenge. Though some cases are caused by highly penetrant mutations in amyloid precursor protein (*APP*) or the presenilins (*PSEN1* and *PSEN2*), components of the enzymatic secretase complex that processes APP to the beta-amyloid peptide, most AD cases are “sporadic” (Alzheimer’s Disease Collaborative 1995; Goate et al. 1991; Lendon et al. 1997; Levy-Lahad et al. 1995; Rogaev et al. 1995). Risk alleles, most notably of *APOE4*, have also been identified (Corder et al. 1993). However, introducing these alleles into the mouse genome, even as overexpressed versions of the human disease-associated mutations, has not fully recapitulated the full scope of Alzheimer’s disease pathology, including memory and cognitive deficits, extracellular beta-amyloid plaques, intracellular neurofibrillary tangles of Tau, and neuronal cell loss in brain regions such as the cortex and hippocampus. The attempted solution to this is to stack together the monogenic variants that lead to Alzheimer’s disease into a single model. Such a model is the 5X familial Alzheimer’s disease mouse (5XFAD), which carries three disease-associated variants in APP and two disease-associated variants found in PSEN1 (Oakley et al. 2006). These mice develop early amyloid accumulation, show synaptic and neuronal loss, and have cognitive deficits, but their genotype is a conglomerate, and does not represent the genome of any one AD patient. Models such as 5XFAD may be useful for studies of the cell biology of plaque formation or the contribution of factors such as neuro-inflammation to neural pathological and cognitive changes, but to date the predictive validity of AD models has been generally poor.

More sophisticated approaches may be necessary to produce more valid AD models. One consideration is the genetic background of the mice. Introducing genetic variability beyond the standard C57BL/6 strain background may create a more permissive and predictive genetic environment. Recent studies suggest that C57BL/6 is in fact a fairly resistant strain for both cognitive problems and histopathological phenotypes in the face of the 5XFAD mutations. Introducing DBA/2J alleles through crosses to recombinant inbred BXD lines created a range of phenotypes, and has the potential to identify interacting loci that affect the outcome (Neuner et al. 2018). Thus, more complex genetics beyond simply introducing the disease-associated variants may be necessary.

In addition to introducing genetic risk factors, environmental risk factors may also influence the validity of models. Such factors include environmental enrichment, diet, and exercise, all of which have been shown to alter AD phenotypes in mice (Graham et al. 2016; Jankowsky et al. 2005). For example, transgenic mice expressing Alzheimer’s-associated APP and PSEN1 transgenes performed better in cognitive tasks when housed in an enriched environment than when housed under standard conditions. Paradoxically, however, the amyloid plaque load was actually increased by environmental enrichment (Jankowsky et al. 2003, 2005). Thus, housing conditions influence both the behavioral and neuropathological phenotypes in these mice, albeit in apparently opposite directions.

Ultimately, the extent to which rodents will serve as good preclinical AD models remains to be determined, and additional preclinical validation between mouse studies and clinical trials may be needed. The application of CRISPR/Cas9 genome editing to non-human primates such as marmosets or mouse lemurs that are also relatively tractable as laboratory models may provide a final preclinical validation. Importantly, the mouse lemur naturally develops an AD-like neurodegeneration, possibly indicating that it will indeed provide a valid model of AD (Izpisua Belmonte et al. 2015).

**Fig. 3:**
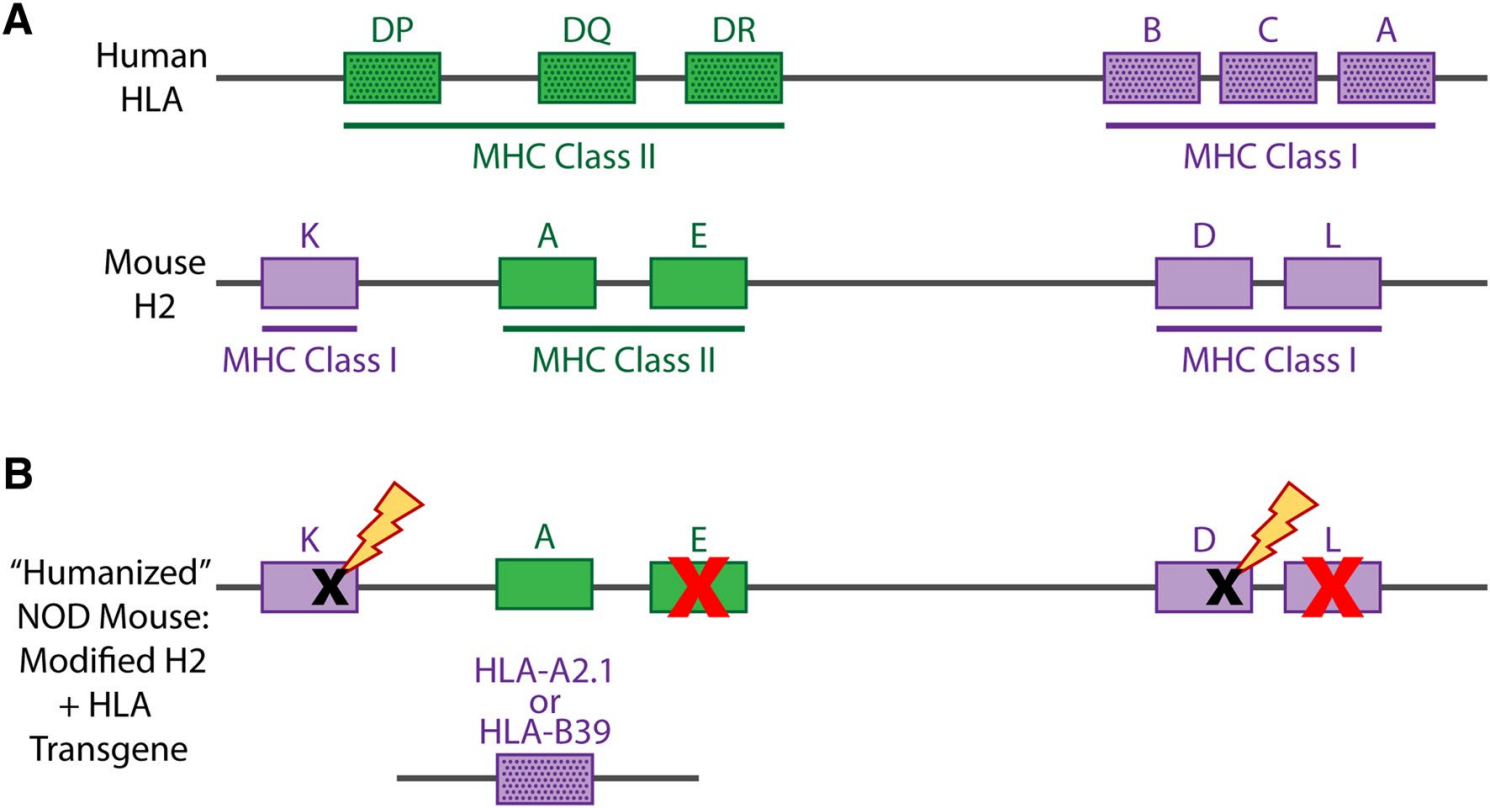
“Humanized” mice for type I diabetes research carry human major histocompatibility complex (MHC) alleles. **a** Schematic of the regions encoding MHC genes: HLA in humans (top) and H2 in mice (bottom). Class I MHC genes are depicted in purple, class II genes in green; human genes are stippled. **b** In “humanized” NOD mice, H2.K and H2.D have been genetically ablated (black “x” plus lightning bolt). NOD mice naturally harbor mutations (red “x”) in H2.E and H2.L, and thus H2.A (class II) is the only functional MHC gene remaining in the H2 locus. Human HLA alleles (class I; HLA.-A2.1 or HLA-.B39) are replaced as a transgene to restore T1D susceptibility to otherwise T1D-resistant, class I-deficient mice

Alzheimer’s is one example of a more complex, multifactorial disease that involves a few known driver loci, many genetic risk loci with varying levels of contribution, and generally poorly defined environmental and lifestyle factors. A somewhat different example is found in autoimmune diseases such as type I diabetes (T1D). The human risk alleles associated with T1D generally identify the major histocompatibility complex (MHC), and mouse models that spontaneously develop diabetes, such as the non-obese diabetic mouse (NOD), have many risk loci for T1D, but again the MHC on chromosome 17 is the strongest association. However, the mouse MHC, termed “H2,” differs significantly from the human equivalent HLA gene complex. A solution is to generate “humanized” mouse models by systematically deleting the mouse MHC genes and replacing them by transgenic copies of the human variants of interest without concern about competition with the endogenous mouse sequences (Racine et al. 2018). Within the MHC, particular unusual class II variants contribute to T1D by mediating autoreactive CD4 T-cell responses. However, in the right genetic context, particular MHC class I molecules, including some common variants, mediate autoreactive CD8 T-cell responses. These CD8 T-cell responses are essential to T1D development in NOD mice, and also likely in humans. Since pancreatic ß-cells express MHC class I, but not class II molecules, autoreactive CD8 T-cells are likely the ultimate mediators of T1D development. Thus, a potential T1D intervention may be to find a way to block the development or functional activation of MHC class I-restricted ß-cell autoreactive CD8 T-cells. To provide models for testing such possible T1D interventions, CRISPR/Cas9 technology was utilized to directly ablate the classical murine MHC class I molecules normally expressed by NOD mice (Kd and Db), which were then replaced with the human disease-associated HLA-A2.1 or -B39 variants (designated NOD-cMHCI^−/−^-A2 and NOD-cMHCI^−/−^-B39 mice) (Fig. 3). HLA-A2 or -B39 expression restores T1D susceptibility to otherwise completely disease-free murine MHC class I-deficient NOD mice (Schloss et al. 2018). The NOD-cMHCI^−/−^-A2 and NOD-cMHCI^−/−^-B39 strains are now being used to test whether diabetogenic CD8 T-cell responses can be attenuated. These HLA haplotypes are of pathological significance to a preponderance (> 60%) of human patients.

In addition to replacing mouse loci with pathogenic human variants, there are several alternative strategies for incorporating the patient genome into the model. The first is xenografting: taking patient-derived tissue and implanting it into an animal model such as a mouse (Walsh et al. 2017). This is been most successfully used in cancer studies, but has been applied to other disease areas as well. While this approach obviously captures the patient’s genetics, it poses some challenges in terms of environment. Tumors, for example, are often engrafted into the flank, and not their original site, potentially impacting stromal interactions and vascularization. Furthermore, to prevent immune rejection of the foreign tissue, engraftment is done in an immune-compromised mouse. However, the role of the immune system in cancer is an ever-increasing field of research that is omitted from such models unless more complicated xenografting to introduce a human immune system is also undertaken. Comprehensively discussing the strengths and weaknesses of xenografting approaches and immune-compromised host strains of mice is beyond the scope of this review, but host strains are discussed in detail in the accompanying review article by Shultz et al. (2019). Additional considerations on the validity and use of these models are discussed in other references (Landgraf et al. 2018; Shultz et al. 2012; Walsh et al. 2017; Williams 2018).

The second strategy for capturing the patient genetics is to simply use the patient’s cells as the model. This approach has gained considerable traction in the recent past thanks to cellular reprogramming to create induced pluripotent stem (IPS) cells (Takahashi and Yamanaka 2006). These cells can be differentiated into many different cell types, thus removing the restriction of simply studying the primary isolated cells or immortalized lines derived from them. In cases where a human-specific gene or a complex rearrangement leads to disease, it may be difficult or even impossible to recreate the genetic abnormality in a model organism. For example, a segmental duplication on human chromosome 15q13-14 leads to a fusion the *CHRNA7* and *FAM7A* genes and is associated with schizophrenia (Riley et al. 2002). Studying neurons derived from patient IPS cells provides an experimental system that captures this complicated genetic rearrangement. Advances in culture methods, including 3D culture models and organoids that allow cell–cell interactions, should improve the validity of these experimental systems even further. However, limitations remain. Namely, the phenotype of interest needs to manifest in culture. This is likely to occur for inborn errors of metabolism, cellular phenotypes such as lysosomal storage disorders, or conditions for which there are well-validated and predictive biomarkers that can be monitored in vitro. However, in the extreme, a disease such as autism, which is defined entirely based on patient behavior, will not be tractable in an IPS cell model. A second limitation is that the cells remain relatively immature in culture. This is often cited as a challenge for modeling neurological disorders. Neurons markedly change their excitability, complement of receptors and channels, and conductivity during development and with age. This immaturity may present the largest challenge for modeling age-dependent neurodegenerative diseases. In contrast, diseases such as epilepsy that often have a stronger developmental component may be amenable to cell-based modeling. Indeed, multi-electrode array studies reveal increased excitability in the network properties of IPS cell cultures over the course of their differentiation to striatal neurons. This is related to their expression of *KCNQ* channels, and antiepileptic drugs reverse these properties (Telezhkin et al. 2018). In principle, similar approaches could be used in IPS neuron cultures from epileptic patients. This would create a drug testing and screening platform (see for example Stacey et al. 2018). Furthermore, the IPS cell cultures can be compared to primary neurons isolated from precision animal models, and drugs that are successful in vitro can then be tested in vivo. In such a complementary approach, the advantages of IPS cells including assessing target engagement in a human setting and the potential for screening compounds with at least moderate throughput can be combined with an in vivo model. This can confirm that a cellular phenotype, such as reducing multi-electrode array hyper-excitability, translates to a clinically relevant phenotype of interest, such as preventing seizures (Epi 2015; Grainger et al. 2018; Tidball and Parent 2016).

Patient-derived IPS cells are attractive for completely capturing the patient genome, but are also slow and expensive to generate through reprogramming, may carry somatic passenger mutations, and may have intrinsic variability derivation-to-derivation and differentiation-to-differentiation. Perhaps most practically, for rare diseases, identifying and obtaining consent from patients may be limiting. CRISPR/Cas9 genome editing offers an opportunity to engineer variants of interest into existing, well-characterized cell lines. This can be efficient within the usual limits of CRISPR/Cas9. For instance, gene inactivation and introduction of single-nucleotide changes and indels can be quite efficient, and even homozygous changes can be introduced. However, larger chromosomal rearrangements are likely to be more problematic and less efficient. Ideally, the variant of interest could be introduced into multiple starting cell lines to control for the particulars of any one cell line and to allow subsequent studies in a variety of genetic backgrounds.

The predictive validity of cell-based models is hard to determine, as the approach is relatively new and examples that have gone to clinical trials are limited. Toxicological and pharmacokinetic/pharmacodynamic studies can be performed in wild-type animals. However, testing efficacy requires a phenotype that can be corrected, and in some cases, this has only been experimentally tractable and attainable with a cell-based system. It may present a challenge for regulatory agencies to accept efficacy data based only on in vitro studies.

Perhaps the greatest challenges for model validity involve systems in which the anatomy is not conserved. An example of this is macular degeneration, a leading cause of age-dependent blindness that results from the degeneration of the central retina containing the cone photoreceptors used for high acuity vision. The underlying cause of macular degeneration is often dystrophy of the retinal pigment epithelium that immediately surrounds the photoreceptors, providing trophic support and phagocytosis of shed outer segments. The macula is a primate-specific anatomical specialization, but the relationship of photoreceptors and pigment epithelium is conserved in many vertebrates, including mice. Other mammals, such as dogs, have a cone-rich, fovea-like region of the retina termed the area centralis, which may be a surrogate for the macula in humans (Guziewicz et al. 2017; Miyadera et al. 2012). Similarly, mutations in *CTNNA1* that cause butterfly retinopathy in humans, a condition leading to macular dystrophy, cause regional degeneration of photoreceptors in mice (Saksens et al. 2016). These results suggest that domains of the mouse retina may be similarly specialized, though this remains controversial. Nonetheless, the general cell biology of the retinal pigment epithelium (RPE) is conserved, including tight junctions and adherens junctions in the epithelial sheet, and mutations in components of these junctions, such as Crumbs (*CRB1*), lead to RPE dystrophy in both mice and humans (den Hollander et al. 2004; Mehalow et al. 2003).

In this regard, RPE dystrophy in mice may be a “phenolog” of various retinal degenerative conditions in humans, including macular degeneration. A phenolog refers to precisely this situation, in which conserved cell biology leads to relevant phenotypes in model organisms, but these phenotypes lack strong face validity because the anatomical differences preclude an exact recapitulation of the human condition (Robinson and Webber 2014).

## Precisely how precise?

There is no simple answer to how much face validity or construct validity is needed in precision animal models to ensure adequate predictive validity. As the preceding examples illustrate, this ultimately depends on the specific question being addressed. If one wants to test seizure suppression, the model needs to produce seizures, though the precise mutation may not matter. If one wants to test molecular chaperones, the mutation and protein product need to be accurate, but the phenotype may be less critical. Politically, the greater the concordance with the human condition in both face validity and construct validity, the easier the task of convincing regulatory agencies, clinicians, and patients of the relevance of the preclinical studies. For actual preclinical research such as drug studies, shared pathophysiological mechanisms at the cellular and molecular level may be the most important. Amelioration of the phenotype then represents in vivo target engagement and correction of the pathophysiology, even if the phenotype does not perfectly match the disease. However, demonstrating that the drug target is indeed relevant to the disease in question requires some degree of face validity. For genetic conditions, this is particularly true if the drug is targeting a downstream step to circumvent the mutation or if a compensatory pathway is being targeted and the therapy is not directly trying to bolster the activity of the mutated gene product.

For gene therapy approaches, the target is almost always the mutated gene itself. For gene replacement approaches to restore expression in the face of a loss-of-function mutation, construct validity may be minimally important in terms of the precise genetic lesion (i.e., amino acid change, site of truncation) provided the genetic mechanism is accurately reproduced. In other cases, simply restoring expression of the wild-type gene may not be optimal or even useful. For example, CMTX is caused by mutations in *GJB1*, encoding the hexameric gap junction protein Connexin32 (Bergoffen et al. 1993). The demyelinating neuropathy of null alleles is reproduced by knocking out the mouse *Gjb1* gene, and lenti-viral delivery of wild-type *Gjb1* to Schwann cells is efficacious in treating the demyelinating neuropathy in mouse models (Kagiava et al. 2016; Nelles et al. 1996). However, some point mutations in *GJB1* create dominant-negative alleles which lead to mistrafficking of hexamers containing a mutant subunit (Jeng et al. 2006; Kyriakoudi et al. 2017). In the presence of these alleles, the expression of wild-type *GJB1* may be ineffective, as it is not trafficked to the cell membrane. Instead it may actually contribute additional ER stress and be deleterious. In such cases, a solid understanding of the genetic mechanism and cell biology is needed, and multiple models may be required to capture the range of pathophysiology associated with multiple alleles within a single human gene.

Newer approaches in gene therapy such as the use of CRISPR to target gain-of-function alleles such as repeat expansions are likely to require very precise construct validity to move beyond proof-of-concept studies and into actual preclinical tests. One proposed approach is to develop and validate a series of very specific guide RNAs to common single-nucleotide polymorphisms in relevant regions of the human genome, such as sequences surrounding exon one of the Huntington’s gene, which harbors pathogenic CAG expansions (Monteys et al. 2017). Phased genome sequencing of Huntington’s patients would then determine which of these guides would mediate the excision of the expansion, while leaving the healthy allele on the other chromosome intact. Testing such an approach in vitro could be accomplished using patient cells. However, demonstrating that relevant cell types can be targeted with adequate efficiency in vivo will require models in which the human genome sequence is precisely reproduced, essentially perfect construct validity. Whether in vivo models will be needed for every variant to be targeted is an interesting consideration. Hopefully, in vitro assays can be used to show efficacy and specificity for most guide RNAs, and in vivo efficiency can be demonstrated for a representative handful, and extrapolated to the rest, but again, this is relatively uncharted territory for regulatory agencies.

## Summary

**Fig. 4:**
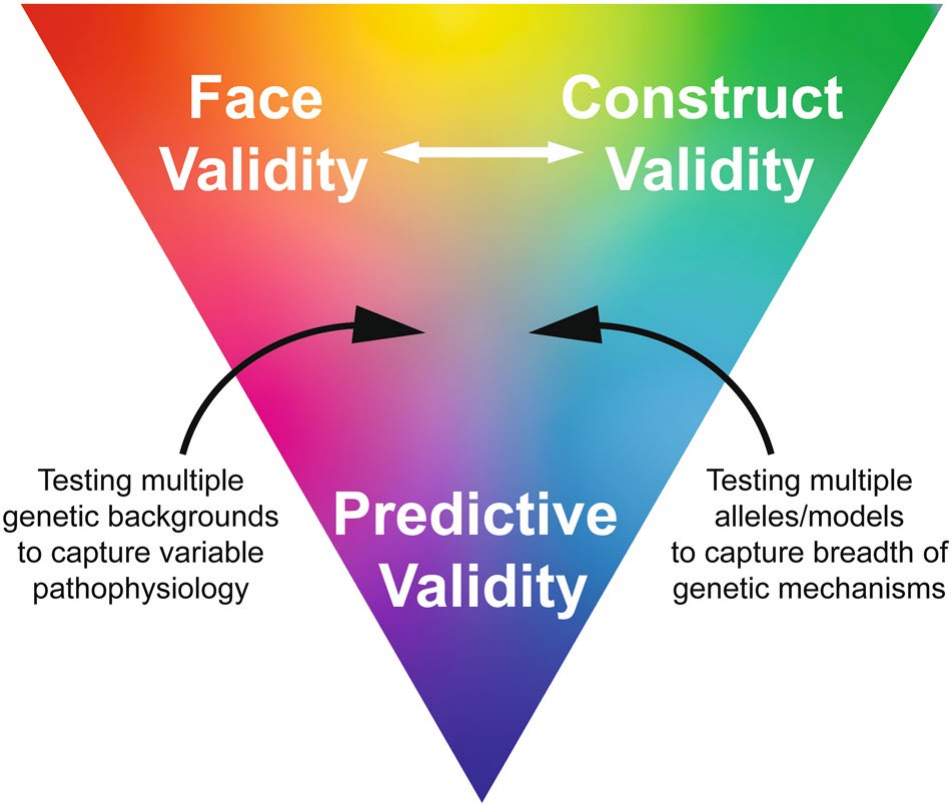
How the predictive validity of precision models can be improved depends on the application. Using multiple genetic backgrounds may better capture the breadth of pathophysiological pathways in humans, and examining multiple disease-associated alleles may capture the breadth of genetic mechanisms, possibly improving the predictive validity and defining the range of treatment responses anticipated in patients

The unprecedented ability to manipulate eukaryotic genomes has created an opportunity to more precisely model a wide variety of human genetic conditions. However, it remains a challenge to model every disease-associated variant, and is likely unnecessary for most preclinical applications. Provided the pathophysiology is reproduced at the molecular level and that the genetic mechanism is reproduced either through genome editing and engineering or through serendipitous spontaneous or induced mutations, the model can be considered to have good construct validity. For many preclinical studies, this may be more important than precise face validity, which in some cases, such as macular degeneration, may be effectively unattainable. However, even for monogenic diseases, capturing the spectrum of genetic mechanisms may require multiple models reproducing different pathogenic alleles (Fig. 4). Predictive validity, the ultimate goal of preclinical work, may be further enhanced by testing therapies in diverse genetic backgrounds that more closely reflect the outbred human population and, therefore, may better capture the breadth of pathophysiology seen in patients. This may be particularly true in more complex diseases, where other risk loci in the genetic background may contribute to the disease or to the response to treatment. Weighing where the precision is needed in developing these models depends on the context of their future applications, and even models that do not perfectly reproduce the human condition may not be so wrong as to not be useful.

## References

[CR1] Achilli F, Bros-Facer V, Williams HP, Banks GT, AlQatari M, Chia R, Tucci V, Groves M, Nickols CD, Seburn KL, Kendall R, Cader MZ, Talbot K, van Minnen J, Burgess RW, Brandner S, Martin JE, Koltzenburg M, Greensmith L, Nolan PM, Fisher EM (2009). An ENU-induced mutation in mouse glycyl-tRNA synthetase (GARS) causes peripheral sensory and motor phenotypes creating a model of Charcot-Marie-Tooth type 2D peripheral neuropathy. Dis Model Mech.

[CR2] Alzheimer’s Disease Collaborative G (1995). The structure of the presenilin 1 (S182) gene and identification of six novel mutations in early onset AD families. Nat Genet.

[CR3] Antonellis A, Ellsworth RE, Sambuughin N, Puls I, Abel A, Lee-Lin SQ, Jordanova A, Kremensky I, Christodoulou K, Middleton LT, Sivakumar K, Ionasescu V, Funalot B, Vance JM, Goldfarb LG, Fischbeck KH, Green ED (2003). Glycyl tRNA synthetase mutations in Charcot-Marie-Tooth disease type 2D and distal spinal muscular atrophy type V. Am J Hum Genet.

[CR4] Bergoffen J, Scherer SS, Wang S, Scott MO, Bone LJ, Paul DL, Chen K, Lensch MW, Chance PF, Fischbeck KH (1993). Connexin mutations in X-linked Charcot-Marie-Tooth disease. Science.

[CR5] Bogdanik LP, Burgess RW (2011). A valid mouse model of AGRIN-associated congenital myasthenic syndrome. Hum Mol Genet.

[CR6] Box GE, Draper N (1987). Empirical model-building and response surfaces.

[CR7] Burgess RW, Nguyen QT, Son YJ, Lichtman JW, Sanes JR (1999). Alternatively spliced isoforms of nerve- and muscle-derived agrin: their roles at the neuromuscular junction. Neuron.

[CR8] Butchbach ME (2016). Copy number variations in the survival motor neuron genes: implications for spinal muscular atrophy and other neurodegenerative diseases. Front Mol Biosci.

[CR9] Carstea ED, Morris JA, Coleman KG, Loftus SK, Zhang D, Cummings C, Gu J, Rosenfeld MA, Pavan WJ, Krizman DB, Nagle J, Polymeropoulos MH, Sturley SL, Ioannou YA, Higgins ME, Comly M, Cooney A, Brown A, Kaneski CR, Blanchette-Mackie EJ, Dwyer NK, Neufeld EB, Chang TY, Liscum L, Strauss JF, Ohno K, Zeigler M, Carmi R, Sokol J, Markie D, O’Neill RR, van Diggelen OP, Elleder M, Patterson MC, Brady RO, Vanier MT, Pentchev PG, Tagle DA (1997). Niemann-Pick C1 disease gene: homology to mediators of cholesterol homeostasis. Science.

[CR10] Corder EH, Saunders AM, Strittmatter WJ, Schmechel DE, Gaskell PC, Small GW, Roses AD, Haines JL, Pericak-Vance MA (1993). Gene dose of apolipoprotein E type 4 allele and the risk of Alzheimer’s disease in late onset families. Science.

[CR11] Cox GA, Mahaffey CL, Frankel WN (1998). Identification of the mouse neuromuscular degeneration gene and mapping of a second site suppressor allele. Neuron.

[CR12] den Hollander AI, Davis J, van der Velde-Visser SD, Zonneveld MN, Pierrottet CO, Koenekoop RK, Kellner U, van den Born LI, Heckenlively JR, Hoyng CB, Handford PA, Roepman R, Cremers FP (2004). CRB1 mutation spectrum in inherited retinal dystrophies. Hum Mutat.

[CR13] DiVincenzo C, Elzinga CD, Medeiros AC, Karbassi I, Jones JR, Evans MC, Braastad CD, Bishop CM, Jaremko M, Wang Z, Liaquat K, Hoffman CA, York MD, Batish SD, Lupski JR, Higgins JJ (2014). The allelic spectrum of Charcot-Marie-Tooth disease in over 17,000 individuals with neuropathy. Mol Genet Genom Med.

[CR14] Duque SI, Arnold WD, Odermatt P, Li X, Porensky PN, Schmelzer L, Meyer K, Kolb SJ, Schumperli D, Kaspar BK, Burghes AH (2015). A large animal model of spinal muscular atrophy and correction of phenotype. Ann Neurol.

[CR15] Elrick MJ, Pacheco CD, Yu T, Dadgar N, Shakkottai VG, Ware C, Paulson HL, Lieberman AP (2010). Conditional Niemann-Pick C mice demonstrate cell autonomous Purkinje cell neurodegeneration. Hum Mol Genet.

[CR16] Epi PMC (2015). A roadmap for precision medicine in the epilepsies. Lancet Neurol.

[CR17] Finkel RS, Mercuri E, Darras BT, Connolly AM, Kuntz NL, Kirschner J, Chiriboga CA, Saito K, Servais L, Tizzano E, Topaloglu H, Tulinius M, Montes J, Glanzman AM, Bishop K, Zhong ZJ, Gheuens S, Bennett CF, Schneider E, Farwell W, De Vivo DC, Group ES (2017). Nusinersen versus sham control in infantile-onset spinal muscular atrophy. N Engl J Med.

[CR18] Foust KD, Wang X, McGovern VL, Braun L, Bevan AK, Haidet AM, Le TT, Morales PR, Rich MM, Burghes AH, Kaspar BK (2010). Rescue of the spinal muscular atrophy phenotype in a mouse model by early postnatal delivery of SMN. Nat Biotechnol.

[CR19] Gautam M, Noakes PG, Moscoso L, Rupp F, Scheller RH, Merlie JP, Sanes JR (1996). Defective neuromuscular synaptogenesis in agrin-deficient mutant mice. Cell.

[CR20] Gelsthorpe ME, Baumann N, Millard E, Gale SE, Langmade SJ, Schaffer JE, Ory DS (2008). Niemann-Pick type C1 I1061T mutant encodes a functional protein that is selected for endoplasmic reticulum-associated degradation due to protein misfolding. J Biol Chem.

[CR21] Goate A, Chartier-Harlin MC, Mullan M, Brown J, Crawford F, Fidani L, Giuffra L, Haynes A, Irving N, James L (1991). Segregation of a missense mutation in the amyloid precursor protein gene with familial Alzheimer’s disease. Nature.

[CR22] Graham LC, Harder JM, Soto I, de Vries WN, John SW, Howell GR (2016). Chronic consumption of a western diet induces robust glial activation in aging mice and in a mouse model of Alzheimer’s disease. Sci Rep.

[CR23] Grainger AI, King MC, Nagel DA, Parri HR, Coleman MD, Hill EJ (2018). In vitro Models for seizure-liability testing using induced pluripotent stem cells. Front Neurosci.

[CR24] Grohmann K, Schuelke M, Diers A, Hoffmann K, Lucke B, Adams C, Bertini E, Leonhardt-Horti H, Muntoni F, Ouvrier R, Pfeufer A, Rossi R, Van Maldergem L, Wilmshurst JM, Wienker TF, Sendtner M, Rudnik-Schoneborn S, Zerres K, Hubner C (2001). Mutations in the gene encoding immunoglobulin mu-binding protein 2 cause spinal muscular atrophy with respiratory distress type 1. Nat Genet.

[CR25] Guziewicz KE, Sinha D, Gomez NM, Zorych K, Dutrow EV, Dhingra A, Mullins RF, Stone EM, Gamm DM, Boesze-Battaglia K, Aguirre GD (2017). Bestrophinopathy: an RPE-photoreceptor interface disease. Prog Retinal Eye Res.

[CR26] Hua Y, Sahashi K, Hung G, Rigo F, Passini MA, Bennett CF, Krainer AR (2010). Antisense correction of SMN2 splicing in the CNS rescues necrosis in a type III SMA mouse model. Genes Dev.

[CR27] Huxley C, Passage E, Manson A, Putzu G, Figarella-Branger D, Pellissier JF, Fontes M (1996). Construction of a mouse model of Charcot-Marie-Tooth disease type 1A by pronuclear injection of human YAC DNA. Hum Mol Genet.

[CR28] Huze C, Bauche S, Richard P, Chevessier F, Goillot E, Gaudon K, Ben Ammar A, Chaboud A, Grosjean I, Lecuyer HA, Bernard V, Rouche A, Alexandri N, Kuntzer T, Fardeau M, Fournier E, Brancaccio A, Ruegg MA, Koenig J, Eymard B, Schaeffer L, Hantai D (2009). Identification of an agrin mutation that causes congenital myasthenia and affects synapse function. Am J Hum Genet.

[CR29] Izpisua Belmonte JC, Callaway EM, Caddick SJ, Churchland P, Feng G, Homanics GE, Lee KF, Leopold DA, Miller CT, Mitchell JF, Mitalipov S, Moutri AR, Movshon JA, Okano H, Reynolds JH, Ringach D, Sejnowski TJ, Silva AC, Strick PL, Wu J, Zhang F (2015). Brains, genes, and primates. Neuron.

[CR30] Jankowsky JL, Xu G, Fromholt D, Gonzales V, Borchelt DR (2003). Environmental enrichment exacerbates amyloid plaque formation in a transgenic mouse model of Alzheimer disease. J Neuropathol Exp Neurol.

[CR31] Jankowsky JL, Melnikova T, Fadale DJ, Xu GM, Slunt HH, Gonzales V, Younkin LH, Younkin SG, Borchelt DR, Savonenko AV (2005). Environmental enrichment mitigates cognitive deficits in a mouse model of Alzheimer’s disease. J Neurosci.

[CR32] Jeng LJ, Balice-Gordon RJ, Messing A, Fischbeck KH, Scherer SS (2006). The effects of a dominant connexin32 mutant in myelinating Schwann cells. Mol Cell Neurosci.

[CR33] Kagiava A, Sargiannidou I, Theophilidis G, Karaiskos C, Richter J, Bashiardes S, Schiza N, Nearchou M, Christodoulou C, Scherer SS, Kleopa KA (2016). Intrathecal gene therapy rescues a model of demyelinating peripheral neuropathy. Proc Natl Acad Sci USA.

[CR34] Kyriakoudi S, Sargiannidou I, Kagiava A, Olympiou M, Kleopa KA (2017). Golgi-retained Cx32 mutants interfere with gene addition therapy for CMT1X. Hum Mol Genet.

[CR35] Landgraf M, McGovern JA, Friedl P, Hutmacher DW (2018). Rational design of mouse models for cancer research. Trends Biotechnol.

[CR36] Lefebvre S, Burglen L, Reboullet S, Clermont O, Burlet P, Viollet L, Benichou B, Cruaud C, Millasseau P, Zeviani M (1995). Identification and characterization of a spinal muscular atrophy-determining gene. Cell.

[CR37] Lefebvre S, Burlet P, Liu Q, Bertrandy S, Clermont O, Munnich A, Dreyfuss G, Melki J (1997). Correlation between severity and SMN protein level in spinal muscular atrophy. Nat Genet.

[CR38] Lendon CL, Ashall F, Goate AM (1997). Exploring the etiology of Alzheimer disease using molecular genetics. JAMA.

[CR39] Levy-Lahad E, Wasco W, Poorkaj P, Romano DM, Oshima J, Pettingell WH, Yu CE, Jondro PD, Schmidt SD, Wang K (1995). Candidate gene for the chromosome 1 familial Alzheimer’s disease locus. Science.

[CR40] Loftus SK, Morris JA, Carstea ED, Gu JZ, Cummings C, Brown A, Ellison J, Ohno K, Rosenfeld MA, Tagle DA, Pentchev PG, Pavan WJ (1997). Murine model of Niemann-Pick C disease: mutation in a cholesterol homeostasis gene. Science.

[CR41] Lupski JR, de Oca-Luna RM, Slaugenhaupt S, Pentao L, Guzzetta V, Trask BJ, Saucedo-Cardenas O, Barker DF, Killian JM, Garcia CA, Chakravarti A, Patel PI (1991). DNA duplication associated with Charcot-Marie-Tooth disease type 1A. Cell.

[CR42] Lupski JR, Wise CA, Kuwano A, Pentao L, Parke JT, Glaze DG, Ledbetter DH, Greenberg F, Patel PI (1992). Gene dosage is a mechanism for Charcot-Marie-Tooth disease type 1A. Nat Genet.

[CR43] Lutz CM, Kariya S, Patruni S, Osborne MA, Liu D, Henderson CE, Li DK, Pellizzoni L, Rojas J, Valenzuela DM, Murphy AJ, Winberg ML, Monani UR (2011). Postsymptomatic restoration of SMN rescues the disease phenotype in a mouse model of severe spinal muscular atrophy. J Clin Invest.

[CR44] Maddatu TP, Garvey SM, Schroeder DG, Hampton TG, Cox GA (2004). Transgenic rescue of neurogenic atrophy in the nmd mouse reveals a role for Ighmbp2 in dilated cardiomyopathy. Hum Mol Genet.

[CR45] Maselli RA, Fernandez JM, Arredondo J, Navarro C, Ngo M, Beeson D, Cagney O, Williams DC, Wollmann RL, Yarov-Yarovoy V, Ferns MJ (2011). LG2 agrin mutation causing severe congenital myasthenic syndrome mimics functional characteristics of non-neural (z-) agrin. Hum Genet.

[CR46] Maue RA, Burgess RW, Wang B, Wooley CM, Seburn KL, Vanier MT, Rogers MA, Chang CC, Chang TY, Harris BT, Graber DJ, Penatti CA, Porter DM, Szwergold BS, Henderson LP, Totenhagen JW, Trouard TP, Borbon IA, Erickson RP (2012). A novel mouse model of Niemann-Pick type C disease carrying a D1005G-Npc1 mutation comparable to commonly observed human mutations. Hum Mol Genet.

[CR47] Mehalow AK, Kameya S, Smith RS, Hawes NL, Denegre JM, Young JA, Bechtold L, Haider NB, Tepass U, Heckenlively JR, Chang B, Naggert JK, Nishina PM (2003). CRB1 is essential for external limiting membrane integrity and photoreceptor morphogenesis in the mammalian retina. Hum Mol Genet.

[CR48] Mendell JR, Al-Zaidy S, Shell R, Arnold WD, Rodino-Klapac LR, Prior TW, Lowes L, Alfano L, Berry K, Church K, Kissel JT, Nagendran S, L’Italien J, Sproule DM, Wells C, Cardenas JA, Heitzer MD, Kaspar A, Corcoran S, Braun L, Likhite S, Miranda C, Meyer K, Foust KD, Burghes AHM, Kaspar BK (2017). Single-dose gene-replacement therapy for spinal muscular atrophy. N Engl J Med.

[CR49] Mercuri E, Darras BT, Chiriboga CA, Day JW, Campbell C, Connolly AM, Iannaccone ST, Kirschner J, Kuntz NL, Saito K, Shieh PB, Tulinius M, Mazzone ES, Montes J, Bishop KM, Yang Q, Foster R, Gheuens S, Bennett CF, Farwell W, Schneider E, De Vivo DC, Finkel RS, Group CS (2018). Nusinersen versus Sham Control in Later-Onset Spinal Muscular Atrophy. N Engl J Med.

[CR50] Mitsuhashi S, Ohkuma A, Talim B, Karahashi M, Koumura T, Aoyama C, Kurihara M, Quinlivan R, Sewry C, Mitsuhashi H, Goto K, Koksal B, Kale G, Ikeda K, Taguchi R, Noguchi S, Hayashi YK, Nonaka I, Sher RB, Sugimoto H, Nakagawa Y, Cox GA, Topaloglu H, Nishino I (2011). A congenital muscular dystrophy with mitochondrial structural abnormalities caused by defective de novo phosphatidylcholine biosynthesis. Am J Hum Genet.

[CR51] Miyadera K, Acland GM, Aguirre GD (2012). Genetic and phenotypic variations of inherited retinal diseases in dogs: the power of within- and across-breed studies. Mamm Genome.

[CR52] Miyawaki S, Mitsuoka S, Sakiyama T, Kitagawa T (1982). Sphingomyelinosis, a new mutation in the mouse: a model of Niemann-Pick disease in humans. J Hered.

[CR53] Monani UR, Lorson CL, Parsons DW, Prior TW, Androphy EJ, Burghes AH, McPherson JD (1999). A single nucleotide difference that alters splicing patterns distinguishes the SMA gene SMN1 from the copy gene SMN2. Hum Mol Genet.

[CR54] Monteys AM, Ebanks SA, Keiser MS, Davidson BL (2017). CRISPR/Cas9 editing of the mutant huntingtin allele in vitro and in vivo. Mol Ther.

[CR55] Nelles E, Butzler C, Jung D, Temme A, Gabriel HD, Dahl U, Traub O, Stumpel F, Jungermann K, Zielasek J, Toyka KV, Dermietzel R, Willecke K (1996). Defective propagation of signals generated by sympathetic nerve stimulation in the liver of connexin32-deficient mice. Proc Natl Acad Sci USA.

[CR56] Neuner SM, Heuer SE, Huentelman MJ, O’Connell KMS, Kaczorowski CC (2018). Harnessing genetic complexity to enhance translatability of alzheimer’s disease mouse models: a path toward precision medicine. Neuron.

[CR57] Nizzardo M, Simone C, Rizzo F, Salani S, Dametti S, Rinchetti P, Del Bo R, Foust K, Kaspar BK, Bresolin N, Comi GP, Corti S (2015). Gene therapy rescues disease phenotype in a spinal muscular atrophy with respiratory distress type 1 (SMARD1) mouse model. Sci Adv.

[CR58] Oakley H, Cole SL, Logan S, Maus E, Shao P, Craft J, Guillozet-Bongaarts A, Ohno M, Disterhoft J, Van Eldik L, Berry R, Vassar R (2006). Intraneuronal beta-amyloid aggregates, neurodegeneration, and neuron loss in transgenic mice with five familial Alzheimer’s disease mutations: potential factors in amyloid plaque formation. J Neurosci.

[CR59] Patel PI, Roa BB, Welcher AA, Schoener-Scott R, Trask BJ, Pentao L, Snipes GJ, Garcia CA, Francke U, Shooter EM, Lupski JR, Suter U (1992). The gene for the peripheral myelin protein PMP-22 is a candidate for Charcot-Marie-Tooth disease type 1A. Nat Genet.

[CR60] Pearn J (1978). Incidence, prevalence, and gene frequency studies of chronic childhood spinal muscular atrophy. J Med Genet.

[CR61] Racine JJ, Stewart I, Ratiu J, Christianson G, Lowell E, Helm K, Allocco J, Maser RS, Chen YG, Lutz CM, Roopenian D, Schloss J, DiLorenzo TP, Serreze DV (2018). Improved murine MHC-deficient HLA transgenic NOD mouse models for type 1 diabetes therapy development. Diabetes.

[CR62] Raeymaekers P, Timmerman V, Nelis E, De Jonghe P, Hoogendijk JE, Baas F, Barker DF, Martin JJ, De Visser M, Bolhuis PA (1991). Duplication in chromosome 17p11.2 in Charcot-Marie-Tooth neuropathy type 1a (CMT 1a). The HMSN Collaborative Research Group. Neuromuscul Disord.

[CR63] Ratni H, Ebeling M, Baird J, Bendels S, Bylund J, Chen KS, Denk N, Feng Z, Green L, Guerard M, Jablonski P, Jacobsen B, Khwaja O, Kletzl H, Ko CP, Kustermann S, Marquet A, Metzger F, Mueller B, Naryshkin NA, Paushkin SV, Pinard E, Poirier A, Reutlinger M, Weetall M, Zeller A, Zhao X, Mueller L (2018). Discovery of Risdiplam, a Selective Survival of Motor Neuron-2 (SMN2) gene splicing modifier for the treatment of spinal muscular atrophy (SMA). J Med Chem.

[CR64] Riley B, Williamson M, Collier D, Wilkie H, Makoff A (2002). A 3-Mb map of a large Segmental duplication overlapping the alpha7-nicotinic acetylcholine receptor gene (CHRNA7) at human 15q13-q14. Genomics.

[CR65] Robinson PN, Webber C (2014). Phenotype ontologies and cross-species analysis for translational research. PLoS Genet.

[CR66] Rogaev EI, Sherrington R, Rogaeva EA, Levesque G, Ikeda M, Liang Y, Chi H, Lin C, Holman K, Tsuda T (1995). Familial Alzheimer’s disease in kindreds with missense mutations in a gene on chromosome 1 related to the Alzheimer’s disease type 3 gene. Nature.

[CR67] Saksens NT, Krebs MP, Schoenmaker-Koller FE, Hicks W, Yu M, Shi L, Rowe L, Collin GB, Charette JR, Letteboer SJ, Neveling K, van Moorsel TW, Abu-Ltaif S, De Baere E, Walraedt S, Banfi S, Simonelli F, Cremers FP, Boon CJ, Roepman R, Leroy BP, Peachey NS, Hoyng CB, Nishina PM, den Hollander AI (2016). Mutations in CTNNA1 cause butterfly-shaped pigment dystrophy and perturbed retinal pigment epithelium integrity. Nat Genet.

[CR68] Schloss J, Ali R, Racine JJ, Chapman HD, Serreze DV, DiLorenzo TP (2018). HLA-B*39:06 efficiently mediates type 1 diabetes in a mouse model incorporating reduced thymic insulin expression. J Immunol.

[CR69] Schrank B, Gotz R, Gunnersen JM, Ure JM, Toyka KV, Smith AG, Sendtner M (1997). Inactivation of the survival motor neuron gene, a candidate gene for human spinal muscular atrophy, leads to massive cell death in early mouse embryos. Proc Natl Acad Sci USA.

[CR70] Seburn KL, Nangle LA, Cox GA, Schimmel P, Burgess RW (2006). An active dominant mutation of glycyl-tRNA synthetase causes neuropathy in a Charcot-Marie-Tooth 2D mouse model. Neuron.

[CR71] Sereda M, Griffiths I, Puhlhofer A, Stewart H, Rossner MJ, Zimmerman F, Magyar JP, Schneider A, Hund E, Meinck HM, Suter U, Nave KA (1996). A transgenic rat model of Charcot-Marie-Tooth disease. Neuron.

[CR72] Sher RB, Aoyama C, Huebsch KA, Ji S, Kerner J, Yang Y, Frankel WN, Hoppel CL, Wood PA, Vance DE, Cox GA (2006). A rostrocaudal muscular dystrophy caused by a defect in choline kinase beta, the first enzyme in phosphatidylcholine biosynthesis. J Biol Chem.

[CR73] Shultz LD, Brehm MA, Garcia-Martinez JV, Greiner DL (2012). Humanized mice for immune system investigation: progress, promise and challenges. Nat Rev Immunol.

[CR74] Shultz LD, Keck J, Burzenski L, Jangalwe S, Vaidya S, Greiner DL, Brehm MA (2019). Humanized mouse models of immunological diseases and precision medicine. Mamm Genome.

[CR75] Stacey P, Wassermann AM, Kammonen L, Impey E, Wilbrey A, Cawkill D (2018). Plate-based phenotypic screening for pain using human iPSC-derived sensory neurons. SLAS Discov.

[CR76] Sugarman EA, Nagan N, Zhu H, Akmaev VR, Zhou Z, Rohlfs EM, Flynn K, Hendrickson BC, Scholl T, Sirko-Osadsa DA, Allitto BA (2012). Pan-ethnic carrier screening and prenatal diagnosis for spinal muscular atrophy: clinical laboratory analysis of > 72,400 specimens. Eur J Hum Genet.

[CR77] Sunada Y, Bernier SM, Kozak CA, Yamada Y, Campbell KP (1994). Deficiency of merosin in dystrophic dy mice and genetic linkage of laminin M chain gene to dy locus. J Biol Chem.

[CR78] Suter U, Welcher AA, Ozcelik T, Snipes GJ, Kosaras B, Francke U, Billings-Gagliardi S, Sidman RL, Shooter EM (1992). Trembler mouse carries a point mutation in a myelin gene. Nature.

[CR79] Takahashi K, Yamanaka S (2006). Induction of pluripotent stem cells from mouse embryonic and adult fibroblast cultures by defined factors. Cell.

[CR80] Telezhkin V, Straccia M, Yarova P, Pardo M, Yung S, Vinh NN, Hancock JM, Barriga GG, Brown DA, Rosser AE, Brown JT, Canals JM, Randall AD, Allen ND, Kemp PJ (2018). Kv7 channels are upregulated during striatal neuron development and promote maturation of human iPSC-derived neurons. Pflugers Arch.

[CR81] Tidball AM, Parent JM (2016). Concise review: exciting cells: modeling genetic epilepsies with patient-derived induced pluripotent stem cells. Stem Cells.

[CR82] Timmerman V, Strickland AV, Zuchner S (2014). Genetics of Charcot-Marie-Tooth (CMT) disease within the frame of the human genome project success. Genes.

[CR83] Verhamme C, King RH, ten Asbroek AL, Muddle JR, Nourallah M, Wolterman R, Baas F, van Schaik IN (2011). Myelin and axon pathology in a long-term study of PMP22-overexpressing mice. J Neuropathol Exp Neurol.

[CR84] Walsh NC, Kenney LL, Jangalwe S, Aryee KE, Greiner DL, Brehm MA, Shultz LD (2017). Humanized mouse models of clinical disease. Annu Rev Pathol.

[CR85] Williams JA (2018). Using PDX for preclinical cancer drug discovery: the evolving field. J Clin Med.

[CR86] Xu H, Wu XR, Wewer UM, Engvall E (1994). Murine muscular dystrophy caused by a mutation in the laminin alpha 2 (Lama2) gene. Nat Genet.

[CR87] Zhao HT, Damle S, Ikeda-Lee K, Kuntz S, Li J, Mohan A, Kim A, Hung G, Scheideler MA, Scherer SS, Svaren J, Swayze EE, Kordasiewicz HB (2018). PMP22 antisense oligonucleotides reverse Charcot-Marie-Tooth disease type 1A features in rodent models. J Clin Invest.

